# Researches on the Cause of the Fluidity of the Blood

**Published:** 1857-04

**Authors:** 


*Researches on the Cause of the Fluidity of the Blood.*—Dr. B. W. Richardson, in a paper read before the chemical section of the British Association for the advancement of science, commenced by giving a historical sketch of the various hypotheses which had been formed to account for the fluidity of the blood and the phenomena of coagulation. He then related his own investigations, which had led him to the discovery that ammonia is a constituent of the blood, and that on its presence the solubility of fibrin, and therefore the fluidity of the blood, is dependent. The numerous experiments performed by the author were described: they may be thus briefly classified: 1. By causing the vapor arising from coagulating blood to pass through another quantity of blood, drawn as nearly as possible at the same time and from the same animal, the coagulation of the latter is suspended so long as the current of vapor is kept up. 2. By driving the vapor of coagulating blood into pure hydrochloric acid, and afterwards treating with chloride of platinum, the characteristic yellow crystals of ammonio-chloride of platinum are procurable. 3. On collecting a large quantity of freshly-drawn blood in a wide-mouthed jar, and placing over a cover, to the interior of which is fixed a slip of glass moistened with hydrochloric acid, the glass becomes covered with microscopic crystals of chloride of ammonium. 4. If fibrin removed from blood be carefully dissolved in a weak solution of ammonia, and again added to the serum and red particles, coagulation may be induced. The result arrived at was, that the phenomenon of coagulation depends essentially on the evolution of ammonia from the blood; and this gives an explanation of the modifications observed in the process of coagulation under various physical conditions. In concluding his paper, Dr. Richardson pointed out that ammonia, in combination with carbonic acid gas, is a constant constituent of the air expired in the breath. The presence of ammonia in the animal economy, and its evolution in respiration, was of interest in that it connected more closely the link that exists between the animal and vegetable worlds. But the subject was of the greatest importance in relation to the causes, the nature, and the treatment of various diseases.—*Proceedings of British Assoc. for Advancement of Sciences*, 1856.

